# A unique MRI-pattern in alcohol-associated Wernicke encephalopathy

**DOI:** 10.1007/s13760-020-01463-7

**Published:** 2020-08-10

**Authors:** Ozan E. Eren, Florian Schöberl, Mattia Campana, Maximilian Habs, Julian Conrad

**Affiliations:** 1 Department of Neurology, University Hospital, LMU, Marchioninistr. 15, Munich, 81377 Germany; 2 Department of Psychiatry, University Hospital, LMU, Nußbaumstraße 7, Munich, 80336 Germany

**Keywords:** Wernicke encephalopathy, Basal ganglia, Vitamin B1 deficiency, Neuroimaging

## Introduction

Wernicke encephalopathy (WE) due to vitamin-B1 (i.e. thiamine) deficiency is an emergency that might easily be overlooked. The most common underlying cause is vitamin-B1 deficiency in the setting of alcohol abuse/addiction. However, there are also many relevant other risk constellations in non-alcoholic patients including inadequate dietary intake (e.g. voluntary starvation, anorexia nervosa, poverty), reduced gastrointestinal absorption (e.g. malignant tumors, after gastric surgery), hyperemesis (e.g. during pregnancy or chemotherapy), decreased hepatic storage or even graft-versus-host disease [1–5]. While the treatment is cheap and readily available in the clinical setting, the failure to recognize a thiamine deficiency can have a detrimental effect on the clinical outcome and the patients’ quality of life [1]. Therefore, knowledge of the clinical and imaging presentation of Wernicke encephalopathy is crucial for clinical practice.

The classical symptoms are central oculomotor disturbances, gait ataxia as well as disorientation and anterograde amnesia. However, the clinical presentation varies and the classical triad of symptoms is rarely present [1]. The typical imaging findings are T2 and T2-Fluid Attenuation Inversion Recovery (FLAIR)-hyperintense lesions of the mammillary bodies, dorsomedial thalami and periaqueductal gray matter [1, 2, 6].

## Case description

Here we present the case of a 40-year-old female alcohol-dependent patient who was assigned from the intensive care unit of an external hospital with the suspected diagnosis of a delir due to long-lasting alcohol consumption (“up to 2 bottles of rum, additionally a few shots of tequila and a couple of beers daily”).

On admission the neurological examination revealed severe disorientation regarding her own age, current location and date. Furthermore, the patient was completely amnestic with a score of 0/3 for verbal recall accompanied by pronounced confabulation and suggestibility. She had jerky eye movements with gaze-evoked nystagmus in all directions and severe gait ataxia with repeated falls.

Because of the patients’ past medical history with ongoing alcohol consumption for many years and the clinical presentation of a malnourished pre-aged female, intravenous vitamin-B1 replacement (500 mg/die) was started immediately with WE as the working diagnosis. Laboratory tests were unremarkable except for a slight to moderate elevation of ferritin (397 ng/ml; normal value 15–150 ng/ml), CRP (1.5 mg/dl; normal value ≤ 1.5 mg/dl), LDH (417U/I; normal value ≤ 249U/I), yGT (119U/I; normal value ≤ 39U/I) and liver enzymes (GOT 105U/I; normal value ≤ 34U/I, GPT 58U/I; normal value ≤ 34U/I). Toxicological screening, cerebrospinal fluid testing and a CT-scan were unremarkable. Unfortunately, vitamin levels were acquired after the first intravenous administration of thiamine.

**Fig. 1:**
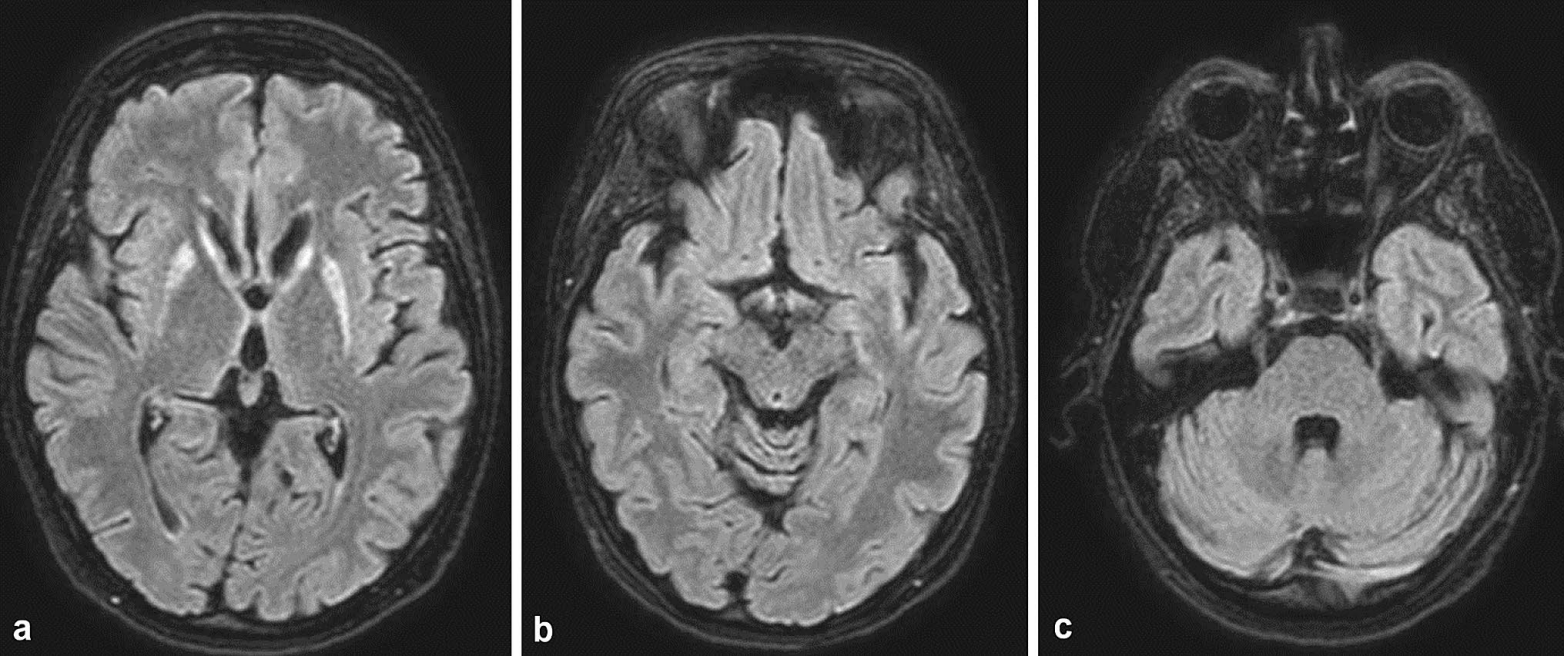
Axial T2-FLAIR shows bilateral hyperintensities in the putamen and caput nuclei caudati (**a**), but sparing (**b** the dorsomedial thalami, periventricular areas around the 3rd ventricle and mesencephalic aqueduct, mammillary bodies)

The MRI-scan revealed a striking pattern with very circumscribed T2-/FLAIR-hyperintensities restricted to the putamen and caput of the caudate nuclei bilaterally (Fig. 1). While we observed the previously described T2-/FLAIR-hyperintensities, no corresponding signal alterations were found in the diffusion-weighted imaging sequences (DWI) and apparent diffusion coefficient maps (ADC). Contrast-enhanced sequences were not acquired in our patient. There was fast and significant improvement of the severe amnesia as well as the accompanying symptoms after starting vitamin-B1 replacement. A follow-up MRI scan was recommended but could not be carried out due to missing consent from our patient.

## Discussion

Until now, the observed MRI-pattern with exclusive symmetric basal ganglia involvement was only described in children and seemed to discriminate pediatric from adult WE [7]. In a case report, two adult patients with malnutrition in the absence of alcohol dependency also showed basal ganglia involvement, in addition to the affection of the typical regions [6, 8]. The characteristic clinical picture and fast response to vitamin-B1 replacement along with the history of the patient confirmed the diagnosis of WE. Particularly, since relevant differential diagnoses with a similar MRI-pattern such as hyper–/hypoglycemia, hypoxia, extrapontine myelinolysis or flavivirus-associated encephalitis could be ruled out [9]. Interestingly, in contrast to the MRI-pattern of bilateral basal ganglia hyperintensities, while completely sparing other brain regions usually affected in WE, the clinical symptoms were not only typical for WE, but they also did not include any extrapyramidal signs.

## Conclusion

WE is a clinical diagnosis and already the suspicion should prompt immediate vitamin-B1 replacement. Our case does not only underpin this important practice in suspected WE, but also illustrates the great diversity of MRI-findings.

## References

[1] Galvin R, Bråthen G, Ivashynka A (2010). EFNS guidelines for diagnosis, therapy and prevention of Wernicke encephalopathy. Eur J Neurol.

[2] Zuccoli G, Cruz DS, Bertolini M (2009). MR imaging findings in 56 patients with Wernicke encephalopathy: nonalcoholics may differ from alcoholics. Am J Neuroradiol.

[3] Di Giuliano F, Picchi E, Scaggiante J (2019). Posterior reversible encephalopathy syndrome and Wernicke encephalopathy in patient with acute graft-versus-host disease. Radiol Case Rep.

[4] Zuccoli G, Gallucci M, Capellades J (2007). Wernicke encephalopathy: MR findings at clinical presentation in twenty-six alcoholic and nonalcoholic patients. Am J Neuroradiol.

[5] Manzo G, De Gennaro A, Cozzolino A (2014). MR imaging findings in alcoholic and nonalcoholic acute Wernicke’s encephalopathy: a review. BioMed Res Int.

[6] Zuccoli G, Siddiqui N, Bailey A, Bartoletti SC (2010). Neuroimaging findings in pediatric Wernicke encephalopathy: a review. Neuroradiology.

[7] Zuccoli G, Pipitone N (2009). Neuroimaging findings in acute Wernicke’s encephalopathy: review of the literature. Am J Roentgenol.

[8] Zuccoli G, Cravo I, Bailey A (2011). Basal ganglia involvement in Wernicke encephalopathy: report of 2 cases. Am J Neuroradiol.

[9] Bekiesinska-Figatowska M, Mierzewska H, Jurkiewicz E (2013). Basal ganglia lesions in children and adults. Eur J Radiol.

